# Long-Term PTSD Risks in Emergency Medical Technicians Who Responded to the 2016 Taiwan Earthquake: A Six-Month Observational Follow-Up Study

**DOI:** 10.3390/ijerph16244983

**Published:** 2019-12-07

**Authors:** Yin Ying Hsiao, Wei Hung Chang, I Chun Ma, Chen-Long Wu, Po See Chen, Yen Kuang Yang, Chih-Hao Lin

**Affiliations:** 1 Department of Psychiatry, National Cheng Kung University Hospital, College of Medicine, National Cheng Kung University, Tainan 70403, Taiwan; ellengarlic@hotmail.com (Y.Y.H.); weihung2364009@gmail.com (W.H.C.); chenps@mail.ncku.edu.tw (P.S.C.); ykyang@mail.ncku.edu.tw (Y.K.Y.); 2 Institute of Clinical Medicine, College of Medicine, National Cheng Kung University, Tainan 70101, Taiwan; 3 Department of Emergency Medicine, National Cheng Kung University Hospital, College of Medicine, National Cheng Kung University, Tainan 70403, Taiwan; momoeunice@yahoo.com.tw; 4 Department of Occupational and Environmental Medicine, National Cheng Kung University Hospital, College of Medicine, National Cheng Kung University, Tainan 70403, Taiwan; clwu@mail.ncku.edu.tw; 5 Institute of Behavioral Medicine, College of Medicine, National Cheng Kung University, Tainan 70101, Taiwan

**Keywords:** posttraumatic stress disorder, emergency medical technician, earthquake, disaster, rescuer, prognostic factor, Taiwan

## Abstract

Although several factors associated with posttraumatic stress disorder (PTSD) in disaster rescue workers were identified in previous studies, the results were inconsistent. This study aimed to explore the prognostic factors of PTSD among disaster rescuers using different screening tools. A 6.4 magnitude earthquake struck southern Taiwan on February 6, 2016. Emergency medical technicians (EMTs) who responded to the earthquake were recruited. The initial survey was conducted one month after the earthquake using a standardized, self-reported, paper-based questionnaire. After six months, we re-evaluated the EMTs using the same questionnaire that was used in the baseline survey. A total of 38 EMT-paramedics were enrolled in the final analysis. Significant differences in PTSD scores at baseline existed between EMTs with and without certain risk factors. The interaction between survey time and risk factors was not significant, but several risk factors correlated with a nonsignificant improvement in the PTSD score after the 6-month follow-up. Perfectionism personality characteristics and several specific field experiences (managing injured patients, managing dead victims, managing dead victims who were pregnant, managing emotionally distraught families, or guilty feelings during the missions) might affect different subdomains of PTSD symptom improvement. Disaster rescuers should be followed up after their missions, regardless of their age, gender, or previous experience with disaster response. EMTs with certain personality characteristics or who are involved in specific field operations should be carefully monitored during and after disaster rescue missions.

## 1. Introduction

Posttraumatic stress disorder (PTSD) could develop when individuals experience life-threatening incidents or major traumatic events [1]. Disaster rescuers who are involved in dangerous missions are at a high risk of developing PTSD [2,3]. PTSD can severely affect both the physical and mental health of professional rescuers. Rescue workers have a pooled current prevalence of PTSD that is significantly greater than that of the general population [4].

Among all occupational groups of rescuers, emergency medical technicians (EMTs) have the highest prevalence of PTSD [5]. EMTs respond to medical emergencies and various types of disasters. Exposure to the dangerous environments and processes inherent in these emergency response missions could lead to psychological stress and result in PTSD. Exposed disaster workers have a high risk of acute stress disorder and PTSD and have a high rate of seeking care for emotional problems [6]. The risk factors of PTSD among EMTs who responded to disasters may include their age, sex, sociodemographic status, cultural background, training levels, and support system [4,7,8,9,10,11,12,13,14].

Among natural disasters, earthquakes are the most capable of causing extensive damage and catastrophic human and economic losses; hence, earthquakes are conceivably among the most traumatizing of events [4]. A survey of rescue workers involved in the 1999 Chi-Chi Earthquake in Taiwan found that disaster rescue work was associated with a high level of stress even for highly trained professionals and may lead to mental health problems [13]. Those who developed PTSD among the rescue workers who responded to the 2008 Wenchuan Earthquake were more likely to have been injured and to have passive coping styles and neurotic personalities [15]. A study that was conducted 8 months after the 2010 Yushu Earthquake demonstrated that middle-aged rescuers and those who had been in serious danger were more likely to develop PTSD symptoms [16]. Involvement in disaster-related work was associated with increased depression and PTSD risks in municipality workers who responded to the 2011 Great East Japan Earthquake [17]. Lower mental function among rescuers in the aftermath of the 2014 Ludian Earthquake was associated with being young, being female, being trapped/in danger, and having a low education level [18].

The importance of mental health among disaster rescuers has been brought to the public’s attention during the last few decades. However, knowledge regarding the relationship between PTSD and personality characteristics, social support, and even the events that rescuers face during their missions is still limited [19,20]. Most of existing reports were derived from cross-sectional studies or follow-up studies using only one evaluation time point. The recruiting objects in most studies were heterogeneous and might involve different levels of rescuers. Furthermore, the risks of PTSD may be associated with the events that disaster rescuers have faced in the field. However, few studies have explored the correlations between field experiences and PTSD.

Gaining an understanding of the potential risk factors of PTSD could enhance mental welfare and reduce potential illness among EMTs following field operations in response to disasters. Information on PTSD risk factors is also valuable for developing appropriate policies governing the occupational safety and psychological health of EMTs during disasters. A 6.4 magnitude earthquake struck southern Taiwan at 03:57 local time on February 6, 2016. The earthquake, which caused 513 injuries and 117 deaths, was the most destructive earthquake to strike Taiwan since the Chi-Chi Earthquake in 1999 [21]. We propose that certain of the risk factors mentioned above may affect the prognosis of PTSD symptoms among specific groups of professional rescuers. This study aimed to identify the possible prognostic factors, including field experiences and other potential risk factors, in EMTs who responded to the disaster.

## 2. Methods

### 2.1. Study Setting

The field operation in response to the 2016 Taiwan earthquake lasted from February 6 to February 14, 2016 [21]. The 13,772 person–day workforce deployed during the 9 day field operation was recorded in the registry system established by the Tainan City Government [22,23]. The field workers included light urban search and rescue teams, hospital-based disaster medical assistance teams, official and volunteer firefighters, and EMTs. EMTs from the Tainan City Government Fire Bureau constituted the majority of the field workers [24].

There were 1024 EMTs affiliated with the Tainan City Government Fire Bureau. Among them, 58 (5.7%) were EMT-basic, 810 (79.1%) were EMT-intermediate, and 156 (15.2%) were EMT-paramedics. A total of 519 EMTs participated in the field search and rescue operation following the earthquake. Of these 519 EMTs, 492 (94.8%) were male. The mean age and work experience were 38 years and 14 years, respectively. Ninety-one (17.5%) of the EMTs were EMT-paramedics, 405 (78.0%) were EMT-intermediate, and 23 (4.4%) were EMT-basic. EMT-paramedics represent the highest training level of EMTs in Taiwan and are usually involved in the most sophisticated missions. Thus, our study focused on EMT-paramedics.

We used a three-step analysis to evaluate PTSD risks in disaster rescuers. First, we attempted to compare PTSD scores between the two groups (with and without risk factors) at baseline. Then, we evaluated the interaction of time and risk factors on PTSD scores to determine the effect of time on PTSD remission. Finally, we compared the PTSD scores between the baseline and the 6-month follow-up. Factors that were associated with nonsignificant improvement of PTSD scores after 6-month follow-up were regarded as poor prognostic factors.

### 2.2. Participants and Data Collection

The Tainan City Government Fire Bureau initiated a program to promote psychological health awareness among EMTs. The program began one month after the earthquake. All participants voluntarily participated in the program. A standardized, self-reported, paper-based questionnaire was distributed and collected 30 min later. To ensure validity and to guide the respondents in rating the degree of their feelings during the previous weeks, consistent brief instructions were provided before the administration of the questionnaire survey. The data from the individual questionnaires were not shared with the Tainan city government and were independently reviewed and analyzed. All of the participants were surveyed again after 6 months of follow-up in a continuing training course for EMT-paramedics.

The survey collected information regarding the demographics of the participants and their field experiences and included the PTSD checklist (PCL) [25]. The standardized questions for field experiences were based on the clinically stressful scenario reported by experienced supervisors of the EMTs. The questions related to previous experiences with field deployment, the date and time of arrival at the disaster field, the overall hours worked in the field, and the main tasks during the mission. Field experiences involving managing injured patients; managing the remains of those who had died, including children (defined as under 8 years old) and women who were apparently pregnant; managing people whose families had died in the disaster; and managing victims’ families who were emotionally distraught were also explored.

### 2.3. Evaluation of PTSD

The PCL comprises 17 typical PTSD symptoms [25,26,27]. Posttraumatic symptoms consist of three separate categories of symptoms: avoidance, re-experience, and hyperarousal. Each individual item is rated as zero or one, with zero representing “never” and one representing “yes”. The category of avoidance includes loss of interest; feeling detached; restricted emotion; avoidance of discussion; avoidance of persons, places or activities; avoidance of recalling the events; and a sense of a foreshortened future. A serious condition of avoidance was defined as having more than three of the symptoms evaluated by the avoidance subscale. The category of re-experience includes flashbacks, nightmares, intrusive recollections, and emotional or physical reactions to triggers. A serious manifestation of re-experience is defined as having more than one of the symptoms evaluated by the re-experience subscale. The category of hyperarousal includes hypervigilance, uncontrolled anger, sleeping difficulty, difficulty concentrating, and an exaggerated startle response. A serious condition of hyperarousal was defined as having more than two of the symptoms from the hyperarousal subscale.

The PCL total score indicated a serious condition when any of the three subscale scores indicated the presence of a serious condition. This method was used in our previous study [28]. The Cronbach’s alpha coefficients reflecting the internal consistency of all three categories were greater than 0.70 in previous studies [29,30] and were greater than 0.75 in our study.

### 2.4. Suicidal Ideation Screening

We used three questions adapted from previous studies to screen for possible suicide risks [28,31]. The three questions were as follows: Do you wish to be dead? Do you want to harm yourself? Do you have feelings of guilt or blame yourself? Because none of the participants answered yes to “Do you wish to be dead?” or “Do you want to harm yourself?”, only the factor “Do you have feelings of guilt or blame yourself?” was analyzed in this study.

### 2.5. Statistical Analysis

The data were analyzed using SPSS version 24.0 (SPSS Inc., Chicago, IL, USA). Descriptive statistics summarizing the characteristics of the study subjects were calculated, including the frequency of the categorical variables and the means and standard deviations (SDs) or medians, ranges, and interquartile range (IQR) of the continuous variables, as appropriate. Two-way ANOVA was performed to test the interaction effect between the prognostic factors of PTSD at baseline and the time factor on the PCL scores. McNemar tests were used to compare the PCL total scores and subscale scores indicating the presence of a serious condition at baseline and the 6-month follow-up. Mann–Whitney U tests were used to test the baseline differences between the presence or absence of prognostic factors. Wilcoxon signed-rank tests were used to evaluate the time effects. The median values of the continuous variables were used as the cutoff values for further comparison. All statistical tests were performed with a two-sided significance level of 0.05.

### 2.6. Ethical Considerations

Data related to individual identification were removed to ensure that the participants remained anonymous throughout the research. The study protocol was reviewed and approved by the Institutional Review Board of National Cheng Kung University Hospital (A-ER-105-267).

## 3. Results

Of the 91 EMT-paramedics who responded to the 2016 Taiwan earthquake, 67 (65 (97.0%) of whom were male) participated in the baseline survey, and 38 EMT-paramedics completed the 6- month follow-up survey. Thus, a total of 38 participants were enrolled in the final analysis. All 38 enrollees were male. The median (range, IQR) age and work experience of the enrollees were 35.0 (28.0–52.0, 7.0) years and 13.5 (6.0–25.0, 7.25) years, respectively. Table 1 presents the demographic data of the enrollees.

Table 2 presents the comparison of the PCL total scores and subscale scores between baseline and the 6-month follow-up. Significant differences were observed between baseline and the 6-month follow-up for the PCL total scores (2.6 ± 3.8 versus 1.5 ± 2.5, *p* = 0.024) and re-experience scores (1.0 ± 1.3 versus 0.4 ± 0.9, *p* = 0.012) but not the avoidance scores or hyperarousal scores (both *p* > 0.05).

Table 3, Table 4, Table 5, Table 6, Table 7 and Table 8 show the differences in the risk factors at baseline and between baseline and the 6-month follow-up. The results showed that, at baseline, the participants who had perfectionism personality characteristics (Mann−Whitney U = −3.13, *p* = 0.002), who managed injured patients (Mann−Whitney U = −2.87, *p* = 0.004), who managed dead victims (Mann−Whitney U = −2.16, *p* = 0.030), who managed dead victims who were pregnant (Mann−Whitney U = −2.35, *p* = 0.019), who managed emotionally distraught families (Mann−Whitney U = −2.89, *p* = 0.004), or who had guilty feelings during the missions (Mann−Whitney U = −3.30, *p* = 0.001) had significantly higher baseline PTSD hyperarousal scores.

At baseline, the EMTs with perfectionism characteristics (Mann−Whitney U = −3.37, *p* = 0.001) and feelings of guilt during the missions (Mann−Whitney U = −2.76, *p* = 0.006) also had significantly higher PTSD total scores. The EMTs with perfectionism characteristics (Mann−Whitney U = −2.67, *p* = 0.008) and rescuers who managed injured victims (Mann−Whitney U = −2.58, *p* = 0.010) had significantly higher baseline PTSD avoidance scores. We did not find significant effects regarding EMT-paramedics’ age, work experience, arrival day, previous deployment experience, or main field mission. The analysis of the other factors can be found in Supplementary Materials.

Furthermore, we did not find a significant effect of the interaction between the prognostic factors of PTSD mentioned above at baseline and the time factor on PCL scores. The time effect was insignificant for the groups with and without prognostic factors. This means that the participants with the prognostic factors of PTSD might show higher posttraumatic stress immediately after the event and remain highly stressed after 6 months of follow-up.

**Figure 1:**
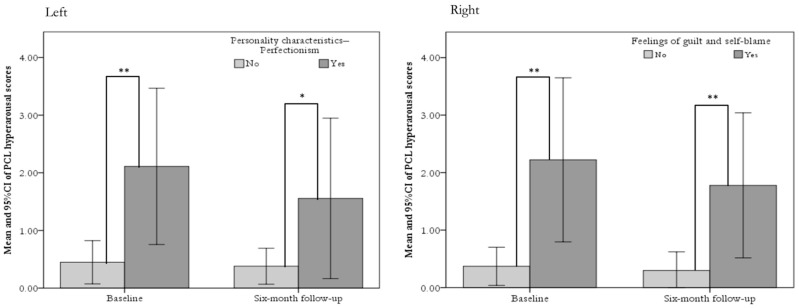
Differences in perfectionism characteristics and guilty feelings between baseline and the 6-month follow-up. Left panel: Perfectionism characteristics and hyperarousal subscale scores. Right panel: Feelings of guilt and self-blame and hyperarousal subscale scores. Note: * indicates *p* < 0.05; ** indicates *p* < 0.01. The error bars indicate the 95% confidence intervals (CIs).

Figure 1 depicts the differences between baseline and the 6-month follow-up with respect to perfectionism characteristics (left panel) and guilty feelings (right panel).

## 4. Discussion

Our study showed that perfectionism personality characteristics and several specific field experiences (managing injured patients, managing dead victims, managing dead victims who were pregnant, managing emotionally distraught families, or experiencing guilty feelings during the missions) might affect different subdomains of PTSD symptom improvement 6 months after a rescue mission. EMTs with these prognostic factors of PTSD exhibit greater posttraumatic stress during the event and remain highly stressed at the 6-month follow-up. EMTs with certain personality characteristics or who are involved in certain field operations should be carefully monitored during and after disaster rescue missions.

Certain risk factors may affect the likelihood of PTSD risk remission [32]. Our findings are in line with previous studies showing that rescuers with perfectionism characteristics may have significantly higher PTSD scores at baseline than those without [33]. Additionally, our findings revealed that subjects who felt guilty and ruminated after a mission also experienced reduced improvement of their PTSD scores [33]. Subjects with perfectionism characteristics ruminate on their failure [34]. Therefore, stressed rescuers with feelings of guilt or failure about their missions are at greater risk of PTSD. Furthermore, biological evidence has shown that perfectionism may be correlated with gray matter volume reduction in the anterior cingulate cortex [35], which is the brain area responsible for emotional regulation and is highly correlated with PTSD [36]. Clinically, rescuers with certain personality tendencies, based on the results of our study, should pay more attention to their psychological condition following rescue missions, and further symptom management can be implemented if the guilty feeling persists.

Stressful rescue missions are correlated with higher PTSD risks. The results of our study further showed that rescuers who were involved in managing dead or injured victims and those who were emotionally upset or whose families had died in the disaster did not see improvements in their PTSD scores after 6 months. It is known that managing death may enhance the risk of PTSD [37], but few studies, if any, have investigated how coping with victims’ families may influence this risk. Our study results implied that the stress experienced while managing aggressive and emotionally distraught families was not less than the stress of managing victims who had died. Our findings resonate with those of previous studies that indicated that stress leading to PTSD among medical staff may result not only from life-threatening medical management but also from the hospital environment and even conflicts with families [38]. Additionally, our study results suggested that managing dead victims who were pregnant may enhance PTSD risks among rescuers. Although no previous studies showed a correlation between PTSD risks and the management of dead victims who were pregnant, a recent study demonstrated that dealing with death and the rescue of children were the most stressful aspects of missions for rescuers [38]. Therefore, the effect of managing dead victims who were pregnant may reflect the combined impact of dealing with death and with the failed rescue of children.

Moreover, those who did not improve significantly after 6 months were mostly impaired in the subdomain of hyperarousal, and subjects with perfectionism characteristics showed a lack of improvement in all the subdomains of PTSD. Although the mechanism of this correlation is unknown, our findings suggest that hyperarousal symptoms indicate the need to follow a rescuer’s condition after a mission. Hyperarousal symptoms could be considered as a main screening tool to monitor the risk of PTSD among disaster rescuers. Additionally, the mental health status of rescuers with perfectionism characteristics should be closely monitored after a mission.

Our study results have important implications for disaster response. Several specific field experiences affect different subdomains of PTSD symptom improvement. During the pre-deployment briefing, the disaster rescuers should be encouraged to understand psychological hazards of certain mission contents and thus, they could be mentally prepared. Although our study did not evaluate the effects of duration and intensity of aforementioned field experiences, we recommend a reasonable rotation of tasks for disaster rescuers. EMTs should avoid overcommitment in certain field tasks for a long period of time. The field camps for EMTs could be geographically and functionally separated from the waiting areas of victims’ families. Another valuable implication of our study is that neither work experience nor age and sex affect the prognosis of PTSD improvement. This is a reminder that more experienced rescuers may not necessarily have a lower risk of developing PTSD [7]. Cumulative stress may increase the risk of PTSD in experienced rescuers [39]. Therefore, disaster rescuers should be monitored after missions, regardless of their age, gender, or previous experience with disaster response [40,41,42].

## 5. Limitations

Our study has several limitations. First, the sample size in this study was small, which may decrease the value of the comparisons. We were unable to analyze the role of sex since all of the study participants were male. Second, we used self-administered questionnaires to assess PTSD symptoms and did not conduct a psychiatric diagnostic interview to confirm the questionnaire results, which may have weakened the value of our study. Third, we did not evaluate the effects of duration and intensity of certain task components in the disaster field. Fourth, there was no information on whether the participants had also joined other missions during the 6-month follow-up, but such experiences may have influenced the PTSD scores. Finally, our study followed participants for only 6 months, and a longer follow-up period would allow further exploration of the long-term impact of PTSD in future studies.

## 6. Conclusions

Our study revealed several risk factors that might affect the prognosis of PTSD risk. Identifying these factors may offer insights for future occupational health programs for rescuers. It is important to be especially aware of those rescuers who have the aforementioned risk factors, as they could be indicators of a poor PTSD prognosis in this group. It is also necessary to arrange both short-term and even long-term counseling programs for stress coping management and intervention for rescue workers, especially for those who have the aforementioned risk factors, in order to reduce their potential psychological trauma after rescue missions and enhance their mental health in future major rescue missions. A study with longer follow-up durations and identifying the different mechanisms of the PTSD subdomains is needed. An understanding of the different mechanisms could lead to the development of better programs to help rescuers and to better treatment strategies when treatment is necessary.

## Figures and Tables

**Table 1:** Demographics of the enrollees (N = 38).

Demographics, Personal Characteristics, and Field Experiences	Data
Age (years) (median, range, IQR)	35.0, 28.0–52.0, 7.0
Sex: male (n, %)	38 (100.0%)
Training level: EMT-paramedic (n, %)	38 (100.0%)
Marital status: married (n, %)	32 (84.2%)
Work experience (years) (median, range, IQR)	13.5, 6.0–25.0, 7.25
Personality: Anxiety (Yes) (n, %)	25 (65.8%)
Personality: Perfectionism (Yes) (n, %)	9 (23.7%)
Personality: Introvert/socially inactive (Yes) (n, %)	14 (36.8%)
Never deployed to disaster field operations (n, %)	18 (47.4%)
Arrived at the field on the first day of the earthquake (n, %)	31 (81.6%)
Hours worked during this field operation (median, range, IQR)	64.0, 8.0–280.0, 54.0
Main tasks performed in the field operation	38 (100.0%)
Search and rescue (n, %)	24 (63.2%)
Emergency medical services (n, %)	14 (36.8%)
Tasks: Managing injured patients (Yes) (n, %)	27 (71.1%)
Tasks: Managing dead people (Yes) (n, %)	27 (71.1%)
Tasks: Managing dead children (under 8 years of age) (Yes) (n, %)	16 (42.1%)
Tasks: Managing dead persons who were apparently pregnant (Yes) (n, %)	5 (13.2%)
Tasks: Managing people whose families had died in the disaster (Yes) (n, %)	26 (68.4%)
Tasks: Managing victims’ families who were emotionally distraught (Yes) (n, %)	21 (55.3%)
Wish to be dead (Yes) (n, %)	0 (0.0%)
Thoughts of self-harm (Yes) (n, %)	0 (0.0%)
Feelings of guilt and self-blame (Yes) (n, %)	9 (23.7%)

Abbreviations: IQR, interquartile range; EMT, emergency medical technician.

**Table 2:** Comparison of posttraumatic stress disorder checklist (PCL) total scores and subscale (re-experience, avoidance, and hyperarousal) scores at baseline and the 6-month follow-up (N = 38).

PCL	Baseline	6-Month Follow-Up	Statistic			
			t	*p*	Wilcoxon Signed-Rank Test	*p*
Total scores						
mean ± SD, range	2.6 ± 3.8, 0–17	1.5 ± 2.5, 0–12	2.36	0.024	−2.26	0.024
serious condition (n, %)	19 (50.0%)	13 (34.2%)				0.109
Re-experience						
mean ± SD, range	1.0 ± 1.3, 0–5	0.4 ± 0.9, 0–3	2.75	0.009	−2.52	0.012
serious condition (n, %)	18 (47.4%)	10 (26.3%)				0.039
Avoidance						
mean ± SD, range	0.8 ± 1.5, 0–7	0.4 ± 1.2, 0–6	1.52	0.138	−1.36	0.174
serious condition (n, %)	4 (10.5%)	3 (7.9%)				1.000
Hyperarousal						
mean ± SD, range	0.8 ± 1.4, 0–5	0.7 ± 1.2, 0–5	1.36	0.181	−1.31	0.190
serious condition (n, %)	8 (21.1%)	6 (15.8%)				0.500

Abbreviation: SD, standard deviation.

**Table 3:** Differences in risk factors at baseline and the differences between baseline and the 6-month follow-up based on the posttraumatic stress disorder checklist (PCL) total scores and subscale (re-experience, avoidance, and hyperarousal) scores. Personality: Perfectionism.

	Baseline Difference between Groups		Interaction between Factors and Survey Time		No (n = 29)						Yes (n = 9)					
					Baseline		6-Month Follow-Up		Difference between Visits		Baseline		6-Month Follow-up		Difference between Visits	
	Mann−Whitney U	*p*	F	*p*	Mean	SD	Mean	SD	Wilcoxon Signed-Rank Test	*p*	Mean	SD	Mean	SD	Wilcoxon Signed-Rank Test	*p*
PCL: total score	−3.37	0.001	4.04	0.052	1.41	2.51	0.83	1.61	−1.31	0.190	6.44	4.77	3.78	3.67	−1.85	0.064
PCL: avoidance	−2.67	0.008	0.88	0.355	0.41	0.95	0.17	0.60	−1.51	0.131	2.00	2.35	1.22	2.05	−0.53	0.596
PCL: hyperarousal	−3.13	0.002	2.43	0.128	0.45	0.99	0.38	0.82	0.00	1.000	2.11	1.76	1.56	1.81	−1.89	0.059

**Table 4:** Differences in risk factors at baseline and the differences between baseline and the 6-month follow-up based on the posttraumatic stress disorder checklist (PCL) total scores and subscale (re-experience, avoidance, and hyperarousal) scores. Tasks: Managing injured patients.

	Baseline Difference between Groups		Interaction between Factors and Survey Time		No (n = 11)						Yes (n = 27)					
					Baseline		6-Month Follow-Up		Difference between Visits		Baseline		6-Month Follow-Up		Difference between Visits	
	Mann−Whitney U	*p*	F	*p*	Mean	SD	Mean	SD	Wilcoxon Signed-Rank Test	*p*	Mean	SD	Mean	SD	Wilcoxon Signed-Rank Test	*p*
PCL: avoidance	−2.58	0.010	1.48	0.232	0.00	0.00	0.09	0.09	−1.00	0.317	1.11	0.33	0.56	0.26	−1.55	0.121
PCL: hyperarousal	−2.87	0.004	0.75	0.392	0.00	0.00	0.00	0.00	0.00	1.000	1.19	0.29	0.93	0.26	−1.31	0.190

**Table 5:** Differences in risk factors at baseline and the differences between baseline and the 6-month follow-up based on the posttraumatic stress disorder checklist (PCL) total scores and subscale (re-experience, avoidance, and hyperarousal) scores. Tasks: Managing dead people.

	Baseline Difference between Groups		Interaction between Factors and Survey Time		No (n = 11)						Yes (n = 26)					
					Baseline		6-Month Follow-up		Difference between Visits		Baseline		6-Month Follow-Up		Difference between Visits	
	Mann−Whitney U	*p*	F	*p*	Mean	SD	Mean	SD	Wilcoxon Signed-Rank Test	*p*	Mean	SD	Mean	SD	Wilcoxon Signed-Rank Test	*p*
PCL: hyperarousal	−2.16	0.030	3.16	0.084	0.18	0.18	0.36	0.28	−1.41	0.157	1.11	0.29	0.78	0.25	−1.93	0.054

**Table 6:** Differences in risk factors at baseline and the differences between baseline and the 6-month follow-up based on the posttraumatic stress disorder checklist (PCL) total scores and subscale (re-experience, avoidance, and hyperarousal) scores. Tasks: Managing dead persons who were apparently pregnant.

	Baseline Difference between Groups		Interaction between Factors and Survey Time		No (n = 33)						Yes (n = 5)					
					Baseline		6-Month Follow-Up		Difference between Visits		Baseline		6-Month Follow-Up		Difference between Visits	
	Mann−Whitney U	*p*	F	*p*	Mean	SD	Mean	SD	Wilcoxon Signed-Rank Test	*p*	Mean	SD	Mean	SD	Wilcoxon Signed-Rank Test	*p*
PCL: hyperarousal	−2.35	0.019	3.34	0.076	0.64	0.22	0.55	0.20	−0.91	0.366	2.20	0.73	1.40	0.68	−1.00	0.317

**Table 7:** Differences in risk factors at baseline and the differences between baseline and the 6-month follow-up based on the posttraumatic stress disorder checklist (PCL) total scores and subscale (re-experience, avoidance, and hyperarousal) scores. Tasks: Managing victims’ families who were emotionally distraught.

	Baseline Difference between Groups		Interaction between Factors and Survey Time		No (n = 17)						Yes (n = 21)					
					Baseline		6-Month Follow-Up		Difference between Visits		Baseline		6-Month Follow-Up		Difference between Visits	
	Mann−Whitney U	*p*	F	*p*	Mean	SD	Mean	SD	Wilcoxon Signed-Rank Test	*p*	Mean	SD	Mean	SD	Wilcoxon Signed-Rank Test	*p*
PCL: hyperarousal	−2.89	0.004	2.74	0.107	0.18	0.13	0.24	0.18	−0.58	0.564	1.38	0.36	1.00	0.31	−1.73	0.084

**Table 8:** Differences in risk factors at baseline and the differences between baseline and the 6-month follow-up based on the posttraumatic stress disorder checklist (PCL) total scores and subscale (re-experience, avoidance, and hyperarousal) scores. Feelings of guilt and self-blame.

	Baseline Difference between Groups		Interaction between Factors and Survey Time		No (n = 27)						Yes (n = 9)					
					Baseline		6-Month Follow-Up		Difference between Visits		Baseline		6-Month Follow-Up		Difference between Visits	
	Mann−Whitney U	*p*	F	*p*	Mean	SD	Mean	SD	Wilcoxon Signed-Rank Test	*p*	Mean	SD	Mean	SD	Wilcoxon Signed-Rank Test	*p*
PCL: total scores	−2.76	0.006	3.89	0.057	1.48	2.19	0.93	1.73	−1.96	0.051	6.11	5.64	3.44	3.81	−1.12	0.261
PCL: hyperarousal	−3.30	0.001	1.31	0.261	0.37	0.84	0.30	0.82	−1.00	0.317	2.22	1.86	1.78	1.64	−0.74	0.461

## Supplementary Materials

The following are available online at https://www.mdpi.com/1660-4601/16/24/4983/s1, Table S1: Age (years). Table S2: Marital status. Table S3. Years of working experience. Table S4. Personality: Anxiety. Table S5. Personality: Perfectionism. Table S6. Personality: Introvert/socially inactive. Table S7. Number of previous deployments to disaster field operations. Table S8. How many days after the earthquake did you arrive at the field? Table S9. How many hours did you participate in the field operation for the 2016 Taiwan earthquake? Table S10. Tasks: Managing injured patients. Table S11. Tasks: Managing dead people. Table S12. Tasks: Managing dead children (under 8 years of age). Table S13. Tasks: Managing dead persons who were apparently pregnant. Table S14. Tasks: Managing people whose families had died in the disaster. Table S15. Tasks: Managing victims’ families who were emotionally distraught. Table S16. Feelings of guilt and self-blame.
