# Supplementary material for: Long-Term PTSD Risks in Emergency Medical Technicians Who Responded to the 2016 Taiwan Earthquake: A Six-Month Observational Follow-Up Study
Source: Int J Environ Res Public Health. 2019 Dec 7;16(24):4983. doi: 10.3390/ijerph16244983 (PMC6950686; doi:10.3390/ijerph16244983)
Supplement: Supplementary file 1 [file ijerph-16-04983-s001.pdf]

Supplementary table: Analysis results for the other factors.

Table S1. Age (years).

	Baseline difference between groups		Interaction between factors and visits		≤35 (n=20)						>35 (n=18)					
					Baseline		6-month follow-up		Difference between visits		Baseline		6-month follow-up		Difference between visits	
	Mann-Whitney U	p	F	p	Mean	SD	Mean	SD	Wilcoxon Signed-Rank Test	P	Mean	SD	Mean	SD	Wilcoxon Signed-Rank Test	P
PCL: total scores	-1.00	0.318	2.90	0.097	3.40	4.64	1.60	3.02	-2.19	0.028	1.72	2.40	1.44	1.98	-0.81	0.416
PCL: re-experience	-0.45	0.656	0.46	0.503	1.10	1.45	0.45	1.00	-2.21	0.027	0.83	1.10	0.44	0.78	-1.31	0.191
PCL: avoidance	-1.28	0.201	3.78	0.060	1.15	1.93	0.35	1.35	-1.78	0.075	0.39	0.78	0.50	0.99	-1.00	0.317
PCL: hyperarousal	-1.29	0.198	1.70	0.200	1.15	1.63	0.80	1.40	-1.40	0.161	0.50	0.99	0.50	0.99	0.00	1.000

Table S2. Marital status.

	Baseline difference between groups		Interaction between factors and visits		Marriage (n=32)						Other than marriage (n=6)					
					Baseline		6-month follow-up		Difference between visits		Baseline		6-month follow-up		Difference between visits	
	Mann-Whitney U	p	F	p	Mean	SD	Mean	SD	Wilcoxon Signed-Rank Test	P	Mean	SD	Mean	SD	Wilcoxon Signed-Rank Test	P
PCL: total scores	-0.34	0.737	2.34	0.135	2.31	2.93	1.53	2.54	-1.80	0.073	4.17	7.05	1.50	2.81	-1.34	0.180
PCL: re-experience	-0.09	0.931	1.15	0.290	0.91	1.09	0.47	0.92	-2.08	0.038	1.33	2.16	0.33	0.82	-1.41	0.157
PCL: avoidance	-0.17	0.865	3.13	0.085	0.69	1.20	0.50	1.27	-0.85	0.398	1.33	2.80	0.00	0.00	-1.34	0.180
PCL: hyperarousal	-0.35	0.728	0.22	0.639	0.72	1.14	0.56	1.01	-0.91	0.366	1.50	2.35	1.17	2.04	-1.00	0.317

Table S3. Years of working experience.

	≤13 (n=19)	>14 (n=19)

	Baseline difference between groups		Interaction between factors and visits		Baseline		6-month follow-up		Difference between visits		Baseline		6-month follow-up		Difference between visits	
	Mann-Whitney U	p	F	p	Mean	SD	Mean	SD	Wilcoxon Signed-Rank Test	P	Mean	SD	Mean	SD	Wilcoxon Signed-Rank Test	P
PCL: total scores	-0.52	0.602	2.51	0.122	3.21	4.69	1.42	1.98	-2.31	0.021	2.00	2.62	1.63	3.06	-0.84	0.404
PCL: re-experience	-0.27	0.787	2.86	0.099	1.11	1.52	0.26	0.56	-2.41	0.016	0.84	1.01	0.63	1.12	-0.92	0.357
PCL: avoidance	-0.44	0.658	1.18	0.284	0.89	1.79	0.26	0.73	-1.51	0.131	0.68	1.25	0.58	1.50	-0.43	0.671
PCL: hyperarousal	-1.52	0.128	0.95	0.337	1.21	1.65	0.89	1.41	-1.19	0.236	0.47	0.96	0.42	0.96	-0.58	0.564

Table S4. Personality: Anxiety.

	Baseline difference between groups		Interaction between factors and visits		No (n=13)						Yes (n=25)					
					Baseline		6-month follow-up		Difference between visits		Baseline		6-month follow-up		Difference between visits	
	Mann-Whitney U	p	F	p	Mean	SD	Mean	SD	Wilcoxon Signed-Rank Test	P	Mean	SD	Mean	SD	Wilcoxon Signed-Rank Test	P
PCL: total scores	-2.04	0.042	2.19	0.148	1.15	2.19	1.00	1.58	-0.55	0.581	3.36	4.25	1.80	2.92	-2.22	0.026
PCL: re-experience	-2.19	0.028	1.25	0.271	0.38	0.87	0.15	0.38	-1.13	0.257	1.28	1.37	0.60	1.04	-2.27	0.023
PCL: avoidance	-1.23	0.217	0.74	0.394	0.23	0.44	0.15	0.38	-1.00	0.317	1.08	1.80	0.56	1.42	-1.24	0.215
PCL: hyperarousal	-1.12	0.261	3.47	0.071	0.54	1.05	0.69	1.11	-1.00	0.317	1.00	1.53	0.64	1.29	-2.06	0.039

Table S5. Personality: Perfectionism.

	Baseline difference between groups		Interaction between factors and visits		No (n=29)						Yes (n=9)					
					Baseline		6-month follow-up		Difference between visits		Baseline		6-month follow-up		Difference between visits	
	Mann-Whitney U	p	F	p	Mean	SD	Mean	SD	Wilcoxon Signed-Rank Test	P	Mean	SD	Mean	SD	Wilcoxon Signed-Rank Test	P
PCL: total scores	-3.37	0.001	4.04	0.052	1.41	2.51	0.83	1.61	-1.31	0.190	6.44	4.77	3.78	3.67	-1.85	0.064

PCL: re-experience	-3.44	0.001	6.31	0.017	0.55	0.91	0.28	0.65	-1.47	0.142	2.33	1.41	1.00	1.32	-2.06	0.040
PCL: avoidance	-2.67	0.008	0.88	0.355	0.41	0.95	0.17	0.60	-1.51	0.131	2.00	2.35	1.22	2.05	-0.53	0.596
PCL: hyperarousal	-3.13	0.002	2.43	0.128	0.45	0.99	0.38	0.82	0.00	1.000	2.11	1.76	1.56	1.81	-1.89	0.059

Table S6. Personality: Introvert/socially inactive.

	Baseline difference between groups		Interaction between factors and visits		No (n=24)						Yes (n=14)					
					Baseline		6-month follow-up		Difference between visits		Baseline		6-month follow-up		Difference between visits	
	Mann-Whitney U	p	F	p	Mean	SD	Mean	SD	Wilcoxon Signed-Rank Test	P	Mean	SD	Mean	SD	Wilcoxon Signed-Rank Test	P
PCL: total scores	-0.24	0.812	0.12	0.735	2.25	0.60	1.29	0.36	-1.81	0.071	3.21	1.33	1.93	0.95	-1.53	0.125
PCL: re-experience	-0.82	0.410	0.56	0.461	0.83	0.24	0.42	0.18	-1.63	0.102	1.21	0.39	0.50	0.25	-1.98	0.047
PCL: avoidance	-0.79	0.429	0.03	0.853	0.75	0.24	0.42	0.18	-1.29	0.196	0.86	0.55	0.43	0.43	-0.54	0.593
PCL: hyperarousal	-0.33	0.739	0.05	0.819	0.67	0.22	0.46	0.18	-0.91	0.366	1.14	0.48	1.00	0.43	-1.00	0.317

Table S7. Number of previous deployments to disaster field operations.

	Baseline difference between groups		Interaction between factors and visits		≥ 1 (n=20)						0 (n=18)					
					Baseline		6-month follow-up		Difference between visits		Baseline		6-month follow-up		Difference between visits	
	Mann-Whitney U	p	F	p	Mean	SD	Mean	SD	Wilcoxon Signed-Rank Test	P	Mean	SD	Mean	SD	Wilcoxon Signed-Rank Test	P
PCL: total scores	-0.43	0.667	0.41	0.528	2.80	3.49	2.00	3.03	-1.01	0.310	2.39	4.20	1.00	1.81	-2.25	0.024
PCL: re-experience	-1.16	0.245	0.02	0.887	1.15	1.23	0.65	1.09	-1.63	0.102	0.78	1.35	0.22	0.55	-1.98	0.047
PCL: avoidance	-0.69	0.489	0.90	0.350	0.90	1.41	0.75	1.55	-0.26	0.796	0.67	1.68	0.06	0.24	-1.84	0.066
PCL: hyperarousal	-0.73	0.466	0.07	0.794	0.75	1.33	0.60	1.10	-0.38	0.705	0.94	1.47	0.72	1.36	-1.41	0.157

Table S8. How many days after the earthquake did you arrive at the field?

	Baseline difference between groups		Interaction between factors and visits		≥2 (n=7)						1 (n=31)					
					Baseline		6-month follow-up		Difference between visits		Baseline		6-month follow-up		Difference between visits	
	Mann-Whitney U	p	F	p	Mean	SD	Mean	SD	Wilcoxon Signed-Rank Test	P	Mean	SD	Mean	SD	Wilcoxon Signed-Rank Test	P
PCL: total scores	-0.49	0.621	0.04	0.833	3.14	6.23	1.86	3.18	-0.54	0.593	2.48	3.15	1.45	2.43	-2.28	0.023
PCL: re-experience	-0.10	0.918	0.06	0.812	1.14	1.86	0.71	1.25	-0.82	0.414	0.94	1.15	0.39	0.80	-2.56	0.010
PCL: avoidance	-0.07	0.945	0.91	0.346	1.29	2.63	0.43	1.13	-0.45	0.655	0.68	1.19	0.42	1.20	-1.21	0.227
PCL: hyperarousal	-1.03	0.305	0.41	0.525	0.71	1.89	0.71	1.89	0.00	1.000	0.87	1.28	0.65	1.05	-1.31	0.190

Table S9. How many hours did you participate in the field operation for the 2016 Taiwan earthquake?

	Baseline difference between groups		Interaction between factors and visits		≤60 (n=17)						>60 (n=20)					
					Baseline		6-month follow-up		Difference between visits		Baseline		6-month follow-up		Difference between visits	
	Mann-Whitney U	p	F	p	Mean	SD	Mean	SD	Wilcoxon Signed-Rank Test	P	Mean	SD	Mean	SD	Wilcoxon Signed-Rank Test	P
PCL: total scores	-0.05	0.962	0.26	0.612	2.88	4.55	2.12	3.35	-0.81	0.419	2.35	3.23	1.10	1.59	-2.22	0.026
PCL: re-experience	-0.40	0.692	0.36	0.552	1.12	1.45	0.71	1.21	-1.21	0.227	0.90	1.17	0.25	0.44	-2.36	0.018
PCL: avoidance	-0.93	0.355	0.01	0.911	1.12	1.96	0.82	1.67	-0.11	0.916	0.45	1.00	0.10	0.31	-1.89	0.059
PCL: hyperarousal	-1.18	0.237	0.48	0.495	0.65	1.41	0.59	1.37	-0.58	0.564	1.00	1.41	0.75	1.12	-0.96	0.336

Table S10. Tasks: Managing injured patients.

	Baseline difference between groups		Interaction between factors and visits		No (n=11)						Yes (n=27)					
					Baseline		6-month follow-up		Difference between visits		Baseline		6-month follow-up		Difference between visits	
	Mann-Whitney U	p	F	p	Mean	SD	Mean	SD	Wilcoxon Signed-Rank Test	P	Mean	SD	Mean	SD	Wilcoxon Signed-Rank Test	P

PCL: total scores	-2.67	0.008	1.27	0.267	0.36	0.20	0.09	0.09	-1.13	0.257	3.52	0.80	2.11	0.54	-2.06	0.039
PCL: re-experience	-1.84	0.066	0.29	0.594	0.36	0.20	0.00	0.00	-1.63	0.102	1.22	0.27	0.63	0.19	-2.12	0.034
PCL: avoidance	-2.58	0.010	1.48	0.232	0.00	0.00	0.09	0.09	-1.00	0.317	1.11	0.33	0.56	0.26	-1.55	0.121
PCL: hyperarousal	-2.87	0.004	0.75	0.392	0.00	0.00	0.00	0.00	0.00	1.000	1.19	0.29	0.93	0.26	-1.31	0.190

Table S11. Tasks: Managing dead people.

	Baseline difference between groups		Interaction between factors and visits		No (n=11)						Yes (n=26)					
					Baseline		6-month follow-up		Difference between visits		Baseline		6-month follow-up		Difference between visits	
	Mann-Whitney U	p	F	p	Mean	SD	Mean	SD	Wilcoxon Signed-Rank Test	P	Mean	SD	Mean	SD	Wilcoxon Signed-Rank Test	P
PCL: total scores	-1.66	0.098	0.75	0.391	1.09	0.58	0.64	0.36	-1.29	0.197	3.22	0.81	1.89	0.55	-1.93	0.053
PCL: re-experience	-1.45	0.146	0.71	0.405	0.55	0.31	0.27	0.27	-1.34	0.180	1.15	0.26	0.52	0.17	-2.22	0.026
PCL: avoidance	-1.15	0.249	0.00	0.990	0.36	0.28	0.00	0.00	-1.34	0.180	0.96	0.33	0.59	0.26	-0.85	0.398
PCL: hyperarousal	-2.16	0.030	3.16	0.084	0.18	0.18	0.36	0.28	-1.41	0.157	1.11	0.29	0.78	0.25	-1.93	0.054

Table S12. Tasks: Managing dead children (under 8 years of age).

	Baseline difference between groups		Interaction between factors and visits		No (n=22)						Yes (n=16)					
					Baseline		6-month follow-up		Difference between visits		Baseline		6-month follow-up		Difference between visits	
	Mann-Whitney U	p	F	p	Mean	SD	Mean	SD	Wilcoxon Signed-Rank Test	P	Mean	SD	Mean	SD	Wilcoxon Signed-Rank Test	P
PCL: total scores	-1.18	0.238	0.30	0.588	2.18	0.86	1.32	0.62	-1.75	0.080	3.19	0.87	1.81	0.49	-1.54	0.124
PCL: re-experience	-0.64	0.520	0.03	0.874	0.86	0.27	0.36	0.20	-2.33	0.020	1.13	0.33	0.56	0.20	-1.44	0.150
PCL: avoidance	-1.19	0.236	0.00	0.982	0.73	0.37	0.36	0.28	-0.81	0.416	0.88	0.30	0.50	0.26	-1.19	0.236
PCL: hyperarousal	-1.85	0.064	2.67	0.111	0.59	0.28	0.59	0.28	0.00	1.000	1.19	0.37	0.75	0.27	-1.51	0.131

Table S13. Tasks: Managing dead persons who were apparently pregnant.

	Baseline difference between groups		Interaction between factors and visits		No (n=33)						Yes (n=5)					
					Baseline		6-month follow-up		Difference between visits		Baseline		6-month follow-up		Difference between visits	
	Mann-Whitney U	p	F	p	Mean	SD	Mean	SD	Wilcoxon Signed-Rank Test	P	Mean	SD	Mean	SD	Wilcoxon Signed-Rank Test	P
PCL: total scores	-2.47	0.013	4.23	0.047	2.12	0.63	1.39	0.45	-1.58	0.114	5.80	1.62	2.40	1.03	-2.04	0.041
PCL: re-experience	-2.66	0.008	5.33	0.027	0.79	0.22	0.42	0.16	-1.75	0.081	2.20	0.37	0.60	0.24	-2.06	0.039
PCL: avoidance	-1.42	0.157	1.03	0.318	0.70	0.26	0.42	0.22	-0.85	0.398	1.40	0.75	0.40	0.24	-1.34	0.180
PCL: hyperarousal	-2.35	0.019	3.34	0.076	0.64	0.22	0.55	0.20	-0.91	0.366	2.20	0.73	1.40	0.68	-1.00	0.317

Table S14. Tasks: Managing people whose families had died in the disaster.

	Baseline difference between groups		Interaction between factors and visits		No (n=12)						Yes (n=26)					
					Baseline		6-month follow-up		Difference between visits		Baseline		6-month follow-up		Difference between visits	
	Mann-Whitney U	p	F	p	Mean	SD	Mean	SD	Wilcoxon Signed-Rank Test	P	Mean	SD	Mean	SD	Wilcoxon Signed-Rank Test	P
PCL: total scores	-1.02	0.307	1.53	0.223	1.33	0.54	1.08	0.56	-0.64	0.524	3.19	0.85	1.73	0.55	-2.24	0.025
PCL: re-experience	-0.80	0.422	0.15	0.703	0.67	0.28	0.25	0.25	-1.09	0.276	1.12	0.27	0.54	0.18	-2.30	0.021
PCL: avoidance	-0.72	0.469	2.32	0.136	0.42	0.23	0.58	0.34	-1.00	0.317	0.96	0.34	0.35	0.23	-1.78	0.075
PCL: hyperarousal	-1.82	0.069	0.85	0.362	0.25	0.18	0.25	0.25	0.00	1.000	1.12	0.31	0.85	0.26	-1.40	0.161

Table S15. Tasks: Managing victims' families who were emotionally distraught.

	No (n=17)	Yes (n=21)

	Baseline difference between groups		Interaction between factors and visits		Baseline		6-month follow-up		Difference between visits		Baseline		6-month follow-up		Difference between visits	
	Mann-Whitney U	p	F	p	Mean	SD	Mean	SD	Wilcoxon Signed-Rank Test	P	Mean	SD	Mean	SD	Wilcoxon Signed-Rank Test	P
PCL: total scores	-2.57	0.010	4.39	0.043	0.94	0.41	0.88	0.41	-0.17	0.864	3.95	0.98	2.05	0.66	-2.49	0.013
PCL: re-experience	-2.19	0.029	1.92	0.174	0.47	0.21	0.24	0.18	-0.85	0.395	1.38	0.31	0.62	0.21	-2.49	0.013
PCL: avoidance	-1.75	0.081	3.46	0.071	0.29	0.17	0.41	0.24	-1.00	0.317	1.19	0.41	0.43	0.29	-1.78	0.075
PCL: hyperarousal	-2.89	0.004	2.74	0.107	0.18	0.13	0.24	0.18	-0.58	0.564	1.38	0.36	1.00	0.31	-1.73	0.084

Table S16. Feelings of guilt and self-blame.

	Baseline difference between groups		Interaction between factors and visits		No (n=27)						Yes (n=9)					
					Baseline		6-month follow-up		Difference between visits		Baseline		6-month follow-up		Difference between visits	
	Mann-Whitney U	p	F	p	Mean	SD	Mean	SD	Wilcoxon Signed-Rank Test	P	Mean	SD	Mean	SD	Wilcoxon Signed-Rank Test	P
PCL: total scores	-2.76	0.006	3.89	0.057	1.48	2.19	0.93	1.73	-1.96	0.051	6.11	5.64	3.44	3.81	-1.12	0.261
PCL: re-experience	-1.75	0.080	1.08	0.307	0.74	1.06	0.33	0.83	-1.93	0.054	1.78	1.72	0.89	1.05	-1.30	0.194
PCL: avoidance	-2.78	0.005	5.06	0.031	0.37	0.84	0.30	0.82	-0.41	0.680	2.11	2.42	0.78	1.99	-1.38	0.168
PCL: hyperarousal	-3.30	0.001	1.31	0.261	0.37	0.84	0.30	0.82	-1.00	0.317	2.22	1.86	1.78	1.64	-0.74	0.461
